# A Case of Limb-Threatening Hematoma in a Patient Taking Citalopram and Apixaban Concurrently

**DOI:** 10.7759/cureus.19771

**Published:** 2021-11-20

**Authors:** Sorabh Datta, Karan Mahal, Shanan Mahal, Angela K Driskill

**Affiliations:** 1 Department of Internal Medicine, Baptist Health–University of Arkansas for Medical Sciences, North Little Rock, USA; 2 Department of Family Medicine, Baptist Health–University of Arkansas for Medical Sciences, North Little Rock, USA

**Keywords:** renal impairment, skin necrosis, limb threatening hematoma, noacs, ssri, citalopram, apixaban, atrial fibrillation, depression

## Abstract

Apixaban, a direct oral anticoagulant, has been demonstrated to increase the risk of bleeding in individuals with atrial fibrillation when used concurrently with citalopram [selective serotonin reuptake inhibitor (SSRI)]. This was proposed as a result of their synergistic anticoagulant effects. We discuss a rare case in which a limb-threatening hematoma was noticed in an 85-year-old female patient who was just begun on citalopram and was on apixaban. There are few published case studies demonstrating a link between these two kinds of drugs, and our case study aims to inform our audience about the possible negative effects of concurrent usage of these two medications.

## Introduction

Apixaban, an oral direct anticoagulant, affects platelet activation and fibrin clot formation by inhibiting both free and clot-bound factor Xa (FXa) in a direct, selective, and reversible manner. Its concomitant use with citalopram [selective serotonin reuptake inhibitor (SSRI)] has been shown to increase bleeding risk in patients with atrial fibrillation [1-3]. This effect is achieved by inhibiting platelet serotonin uptake, probably resulting in synergistic anticoagulant activity. The most frequently encountered hemostatic abnormalities include decreased platelet aggregation and activity as well as an increase in bleeding time. We are presenting a case where apixaban concomitant use with citalopram (SSRI) has caused a limb-threatening hematoma.

## Case presentation

An 85-year-old female patient with a history of persistent atrial fibrillation anticoagulated with oral apixaban 2.5 mg twice daily, depression, and partial nephrectomy presented with a spontaneous left lower extremity hematoma associated with pain and swelling that had been gradually deteriorating over the previous two days. The hematoma was edematous to the point of total skin necrosis. Citalopram was recently started on the patient. No history of trauma or aspirin use was available. The wound measured 30 × 15 cm^2 ^and stretched from the patient's lateral aspect of the tibia to the almost posterior midline of the left calf. The laboratory results revealed normocytic anemia with abnormal coagulation tests. 750 cc of the hematoma was evacuated during surgery using cautery excision of the necrosed skin layers.

## Discussion

A published case report details the occurrence of hemopericardium in two patients who were receiving apixaban together with an SSRI [1]. Serotonin receptors are also located on the surface of platelets. Therefore, greater bleeding with SSRIs may be explained by an augmented pharmacodynamic effect on platelet aggregation inhibition. It has been shown that SSRI and nonvitamin K oral anticoagulants (NOACs) interact pharmacologically through selective inhibition of cytochrome enzymes (CYP2C9) [2,3]. Numerous studies have shown an increased risk of bleeding problems in people with chronic kidney disease. About 27% of apixaban clearance occurs through renal excretion. The remainder is eliminated via the gastrointestinal tract in the form of bile and feces [4-6]. Additionally, concurrent use of SSRIs and apixaban are classified as risk category C, indicating that treatment should be closely watched but may be administered. In this case, the sequential link between developing a spontaneous hematoma becomes critical, particularly given that the only new change in her medical state was the introduction of citalopram (SSRI) [7]. Perhaps when additional reports of severe bleeding associated with apixaban and citalopram become available, this guideline for simultaneous use will be changed to category D (consider modification) or X (avoid combination). Thus the limitation of monitoring NOACs serum drug concentrations also contributes to its low safety profile in these patients.

## Conclusions

The advantages of convenient administration and less frequent monitoring make NOACs more attractive among patients with atrial fibrillation. However, as our knowledge and usage of these treatments expand, we must be vigilant for major life-threatening adverse drug events. Most significantly, as this case indicates, apixaban should be used with great care in individuals with renal impairment and concurrent SSRI treatment.
